# Resistance of vegetation sensitivity to climate and human activities under short-term drought in subtropical humid region: a case study of Guangdong, China

**DOI:** 10.1038/s41598-026-40399-5

**Published:** 2026-02-21

**Authors:** Yuzhen Wu, An Fan, Yuanda Lei, Weishi Xiao, Rumin Wu

**Affiliations:** School of Geographical Sciences, Lingnan Normal University, Zhanjiang, 524048 Guangdong China

**Keywords:** Drought, Vegetation greenness, Subtropical humid region, Vegetation resistance, Climate sciences, Ecology, Ecology, Environmental sciences

## Abstract

Understanding how the changes of vegetation sensitivity to climate and human activities under drought is essential for evaluating ecosystem resistance in subtropical humid regions. This paper focused on Guangdong Province, China, and used SPEI-3 (Standardized Precipitation Evapotranspiration Index) to identify short-term drought events (< 6 months). LMG (Lindeman-Merenda-Gold method) and XGBoost (Extreme Gradient Boosting) were used to quantitatively analyze the contributions of temperature, precipitation and nighttime light (NTL) to the NDVI of evergreen forest, grassland and urban region. LMM (Linear Mixed Model) with month and year as random effects were applied. The conclusions revealed that: (1) Short-term drought significantly reduced the sensitivity of vegetation to temperature in the south of the Tropic of Cancer, while vegetation in the north of the Tropic of Cancer maintained a stable relationship with temperature. (2) Under drought, the temperature contribution to evergreen forest decreased the most (10–13%), followed by urban region (6–15%) and grassland (7–12%). The precipitation contribution to evergreen forest increased by 7–10% and 2–7% for grassland. (3) Drought weakened the positive temperature-NDVI correlation in the west located in the south of the Tropic of Cancer, while the positive temperature-NDVI correlation persisted in the north located in the north of the Tropic of Cancer. (4) Under drought, vegetation maintained a stable positive response to climate. The temperature-NDVI relationship in more than half of the cities shifted from positive to negative.

## Introduction

Under the context of climate change, the frequency of drought events has increased globally leading to negative impact on ecosystem, agriculture and society^1,2^. Researches have indicated that drought influences photosynthesis, net primary productivity and vegetation greenness by altering soil moisture and evapotranspiration, finally impacting ecosystem function^3,4^. Therefore, the impact of drought on ecosystem has become one of the hot topics in global ecological change.

Short-term drought is characterized by high frequency, sudden onset and rapid accumulation of impact. A sharp reduction in water availability or extreme high temperature over a short period often leads to fluctuation in vegetation growth and triggers rapid responses of ecosystem^5^. In recent years, increasing attention has been paid to the impact of short-term drought on vegetation. Researches have indicated that different vegetation types exhibit different responses to short-term water stress and extreme heat due to variations in root structure, physiological mechanism and adaptive strategy^6^. For example, shallow-root vegetation is generally more sensitive to drought^7^as water deficit can lead to reduced photosynthetic rate and leaf discoloration, while deep-root vegetation tends to exhibit a certain degree of resistance due to their stronger water-buffering capacity^8^. Therefore, it is essential to explore the responses of different vegetation types to drought.

Currently, most related researches tend to focus on vegetation changes in traditional arid regions with relatively few studies on the impact of short-term drought on different vegetation types in subtropical humid regions. In fact, the ecosystem in subtropical humid region is quite vulnerable. On one hand, the frequency of drought in this region is increasing^9,10^. On the other hand, the response pattern of vegetation to drought may differ significantly from those in arid regions due to regional climate, human activity pattern and vegetation phenology^11,12^. For example, drought is more likely to disturb soil moisture in subtropical region and further influence vegetation productivity due to the sensitivity of vegetation to soil moisture^13^. Therefore, further research on the response patterns of different vegetation types to short-term drought in subtropical humid region contributes to assessing the ecological stability of this region under extreme climatic condition and improving the understanding of ecosystem mechanism in different climate zones. Existing studies have analyzed the sensitivity of vegetation to climatic factors^14–17^. However, the impact of human activities on vegetation cannot be ignored and may even become the dominant factor in densely populated regions. Human disturbances vary across different land cover types. For example, vegetation in urban regions is generally more susceptible to human impact than that in forests^18,19^. When drought occurs, not only does the impact of climate on vegetation change, but drought-induced alterations in human activities may also affect vegetation compared to normal conditions^20,21^. Therefore, considering different levels of human disturbance, it is necessary to conduct a more detailed classification based on land cover types. In addition, comparing the impacts of climate and human activities on different vegetation under drought and normal conditions is essential for a better understanding of vegetation drought resistance and its driving factor.

Therefore, this paper focused on Guangdong Province, located in a subtropical humid region in China, and used the SPEI (Standardized Precipitation Evapotranspiration Index) to identify drought events with particular emphasis on short-term drought lasting less than 6 months. Although vapor pressure deficit (VPD) and soil moisture are important factors, due to the small spatial resolution (1 km) used in this paper and the larger spatial resolution of existing soil moisture data as well as the fact that temperature and precipitation can reflect some of the evapotranspiration demand and water supply information, this paper focuses on temperature and precipitation as the primary climate factors. The impacts of temperature, precipitation and human activities on three typical land cover types (evergreen forest, grassland and urban region) were quantitatively analyzed using LMG (Lindeman-Merenda-Gold method) contribution analysis, XGBoost (Extreme Gradient Boosting) and LMM (Linear Mixed Model). The spatial heterogeneity of vegetation responses was also studied. This paper explored the following issues: (1) Explore the resistance of sensitivity of vegetation to climate and human activities under short-term drought, and whether there are different response patterns between the northern and southern regions of the Tropic of Cancer. (2) How does short-term drought reshape the sensitivity of vegetation across different land cover types? This paper contributes to a deeper understanding of the response mechanisms of subtropical ecosystem to sudden climatic disturbance and provides a scientific basis for climate adaptation management and environmental impact assessment of human activities.

## Materials and methods

### Study region

**Fig. 1:**
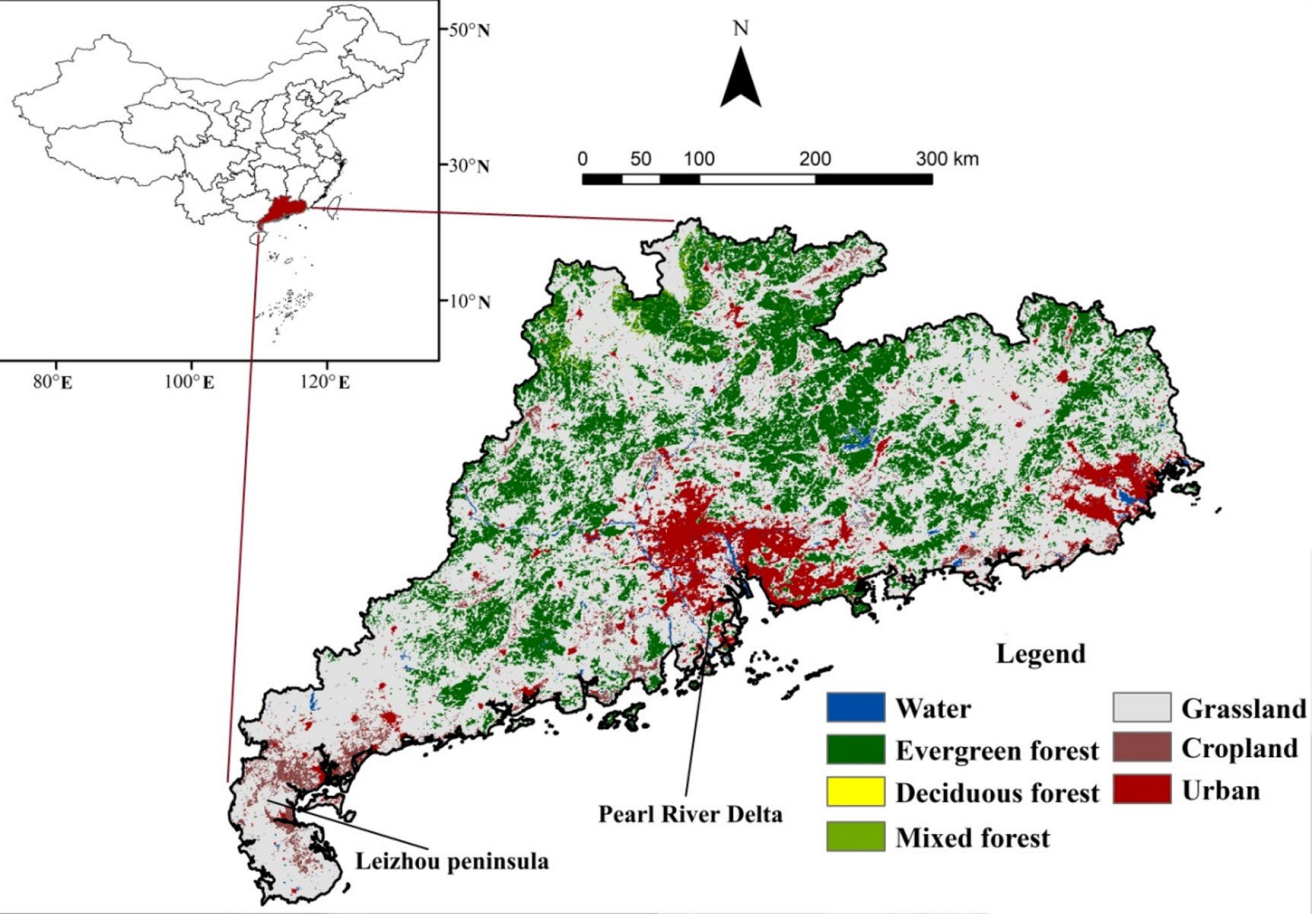
The distribution of land cover types in Guangdong in 2022.

Guangdong Province is located in the southern part of China with the Tropic of Cancer traversing the central region (Fig. 1). The study region is situated in subtropical monsoon climate zone with annual temperature of around 21℃ and precipitation exceeding 1800 mm^22^. Evergreen forest, mixed forest, grassland, cropland and urban region are the dominant land cover types which together account for over 97.9% of the land in Guangdong^23^. Evergreen forest is mainly distributed in the northern and eastern regions, while urban region is primarily in the southern region with the Pearl River Delta urban agglomeration located in the central-southern region. Grassland is widely distributed. In recent years, due to the implementation of ecological policies, forest areas have increased significantly^24^.

### Data and preprocessing

NDVI (Normalized Difference Vegetation Index) is a widely used index for evaluating vegetation greenness. The NDVI used in this paper directly was derived from the MODIS MOD13A3v061 monthly NDVI product (https://lpdaac.usgs.gov) from 2012 to 2022 with a spatial resolution of 1 km. This data has been commonly used in relevant studies and has been proven to be of high quality. Therefore, this paper followed the method of Bai et al.^25^ and Cheng et al.^26^ by directly using this data.

Temperature data was derived from the MODIS MOD11A2v061 8-day product with a spatial resolution of 1 km during 2012–2022 (https://lpdaac.usgs.gov). This dataset is widely used and the original data was directly used^27^. First, all complete 8-day original temperature products within each month were identified. For the products spanning two months, the 8-day temperature value was uniformly assigned to each corresponding day, and the monthly mean temperature was then calculated by averaging all daily values. Further, the potential evapotranspiration data used to calculate SPEI (Standardized Precipitation Evapotranspiration Index) was derived from the MODIS MOD16A2 8-day product (https://lpdaac.usgs.gov) which is commonly used in evapotranspiration research, and the original data was directly used^28^. The spatial resolution is 500 m and it was then interpolated to 1 km using bilinear interpolation. First, all complete 8-day original evapotranspiration products within each month were identified. For the products spanning two months, the 8-day values were allocated to the corresponding month in proportion to the number of days and the monthly potential evapotranspiration was obtained by summing all daily values. Precipitation data were sourced from the 1-km monthly precipitation dataset for China (1901–2022). It was evaluated by the observations from 496 weather stations across China^29,30^. The spatial resolution is 0.0083333° and precipitation data from 2012 to 2022 were selected. To maintain consistent spatial resolution, bilinear interpolation was used to resample the monthly precipitation data to a spatial resolution of 1 km.

Nighttime light data (NTL) is commonly used to represent the intensity of human activities and is widely applied in studies of the impact of human activities on the environment^31,32^. The NTL is sourced from the widely used and high-quality VNP46A3 monthly product from 2012 to 2022 (https://lpdaac.usgs.gov) with a spatial resolution of 15 arc seconds, which was bilinearly interpolated to 1 km.

The land cover data is sourced from the MODIS MCD12Q1 v061 product (https://lpdaac.usgs.gov) with a spatial resolution of 500 m. The data were resampled to 1 km using the nearest neighbor method. This dataset is one of the commonly used datasets for studying land use changes^33^. Further, the 14 original land types based on UMD (University of Maryland) were reclassified into 6 categories with a focus on land cover types with larger areas (Table 1).

**Table 1:** The original and reclassified land cover types.

Original land cover type (UMD)	Reclassified land cover type
Evergreen needleleaf forest, evergreen broadleaf forest	Evergreen forest
Deciduous needleleaf forest, deciduous broadleaf forest	Deciduous forest
mixed forest	Mixed forest
Grasslands, savannas, woody savannas, closed shrubland, open shrubland	Grassland
Water	Water
Cropland	Cropland
Urban and built-up, barren	Urban

### Analytical approach

**Fig. 2:**
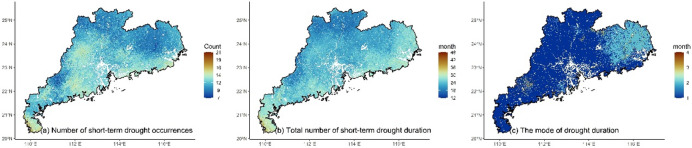
The spatial distribution of drought characteristics based on SPEI-3short-term.

SPEI (Standardized Precipitation Evapotranspiration Index) is a widely used index for drought monitoring. It combines precipitation and potential evapotranspiration to provide a more accurate assessment of drought events. The widespread application of SPEI in drought research has made it an important tool for quantifying drought attribution and evaluating the impacts of climate change^34,35^. SPEI-3 represents the drought conditions of meteorological factors over a 3-month time window, making it suitable for assessing the impact of short-term climate anomalies on ecosystem. Studies have shown that SPEI3 can reveal the relationship between short-term water stress and vegetation growth^36,37^, and it has been extensively used to identify short-term drought^38^. Therefore, this paper used SPEI-3 to identify short-term drought event in Guangdong. A drought event was considered when SPEI-3 is less than − 0.5^39,40^. A drought duration of 6 months is a typical boundary between long-term and short-term droughts which helps avoid confusing the potentially different mechanisms underlying short-term and long-term droughts. In both the National Integrated Drought Information System (NIDIS, https://www.drought.gov/what-is-drought) and NOAA Climate Prediction Center (CPC, https://www.cpc.ncep.noaa.gov/products/international/drought) operational monitoring, a 6-month duration is used as the boundary between short-term and long-term droughts. This boundary is also found in the studies of Awange et al.^41^, Sheffield and Wood^42^. Further, as with the conclusions of Sheffield and Wood^42^, the most common droughts in Guangdong located in the subtropics were also short-term droughts (< 6 months, Fig. 2c). Therefore, a drought event lasting less than 6 months was considered as short-term drought in this paper. The calculation of SPEI can be referenced in the paper by Vicente-Serrano et al. ^43^.

The linear LMG (Lindeman-Merenda-Gold method) is used to identify the contributions of different impact factors to vegetation. LMG quantifies the contributions of independent variables to the dependent variable by decomposing the R² of the linear model. It provides a comprehensive evaluation of the average contribution of independent variable accounting for correlation or multicollinearity among them^44^. The calculation of LMG can be referenced in the paper by Grömping et al.^45^. The higher the LMG value, the higher the contribution of the independent variable.

To further analyze the nonlinear effects of different impact factors on vegetation greenness, XGBoost (Extreme Gradient Boosting) was used to assess the importance of independent variables. XGBoost is a highly efficient and flexible machine learning algorithm that is suitable for complex regression problems. It can handle nonlinear relationship and multiple interaction within the data, enhancing the accuracy by combining the advantage of gradient boosting algorithms^46,47^. This method has been applied in environmental and ecological fields, such as predicting the impacts of environmental factors on vegetation growth and handling complex ecological data^48,49^. The calculation of XGBoost can be referenced in the paper by Chen and Guestrin^50^.

Additionally, LMM (Linear Mixed Model) was used to analyze the relationship among temperature, precipitation, NTL and NDVI. LMM incorporates random effect into a linear regression model to separate within-group and between-group differences^51^. This allows for more accurate capture of complex relationship within the data and a better assessment of the relationship between independent and dependent variables. The method has been applied in environmental and ecological fields^52,53^. To comprehensively explore the relationship among climate, human activities and vegetation greenness under multi-year and short-term drought conditions, this paper set two scenarios with month and year as random effects respectively to evaluate the stable fixed effect of impact factors. The calculation of LMM can be referenced in the paper by Gumedze and Dunne^54^.

Further, in order to exhibit the spatial patterns of the results, this paper used kernel density estimation for presentation.

## Results

### The contribution of climate and human activities to vegetation greenness across different land cover types and corresponding patterns

The high-frequency regions of short-term drought (< 6 months) were mainly located in Leizhou Peninsula, central, western and eastern coastal regions of Guangdong (Fig. 2a). The cumulative duration of short-term drought ranged from12 to 48 months with higher values located in Leizhou Peninsula, eastern and western regions (Fig. 2b). The most common droughts in Guangdong were those with a duration of less than 4 months (Fig. 2c). The mode of drought duration in most areas was 1 month, while it ranged from 2 to 3 months in the eastern and some western regions.

**Fig. 3:**
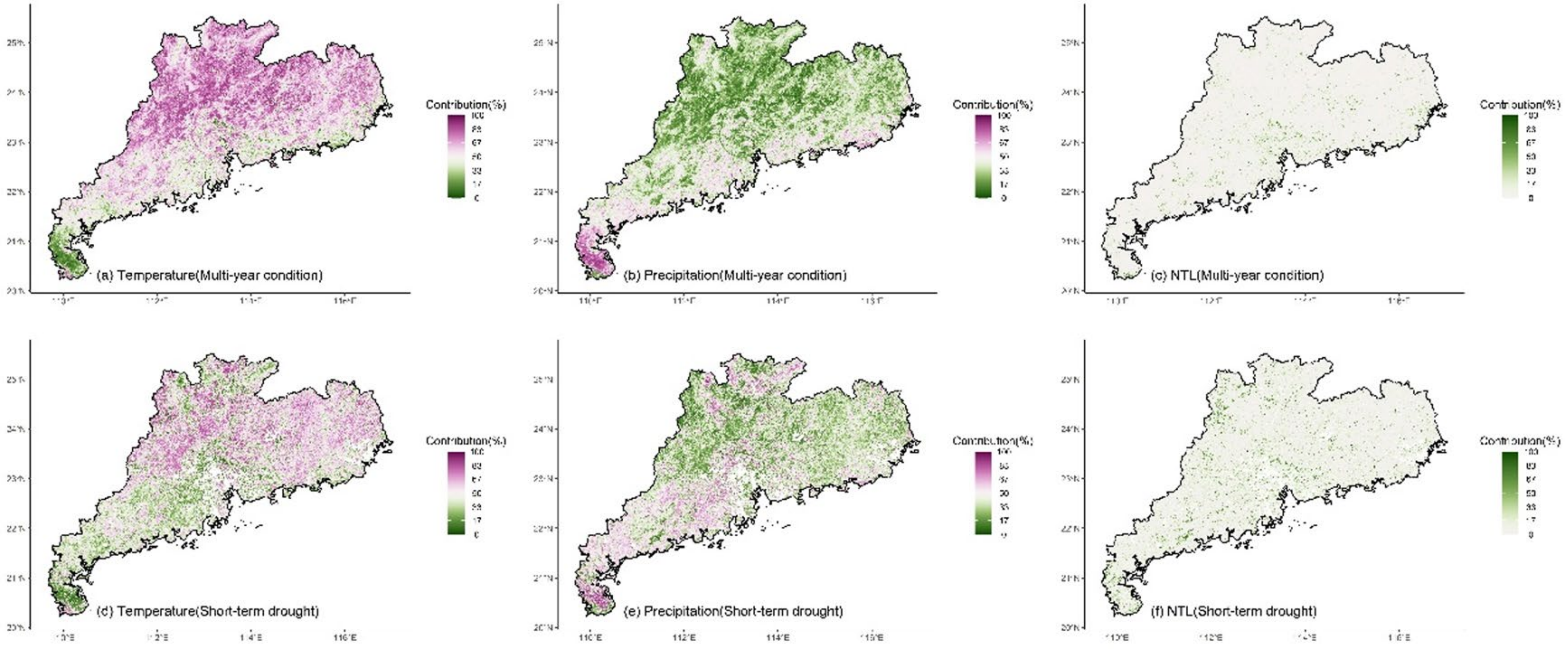
The contribution of climate and human activities to NDVI under multi-year and short-term drought conditions. For each grid, temperature contribution + precipitation contribution + NTL contribution = 100%.

**Fig. 4:**
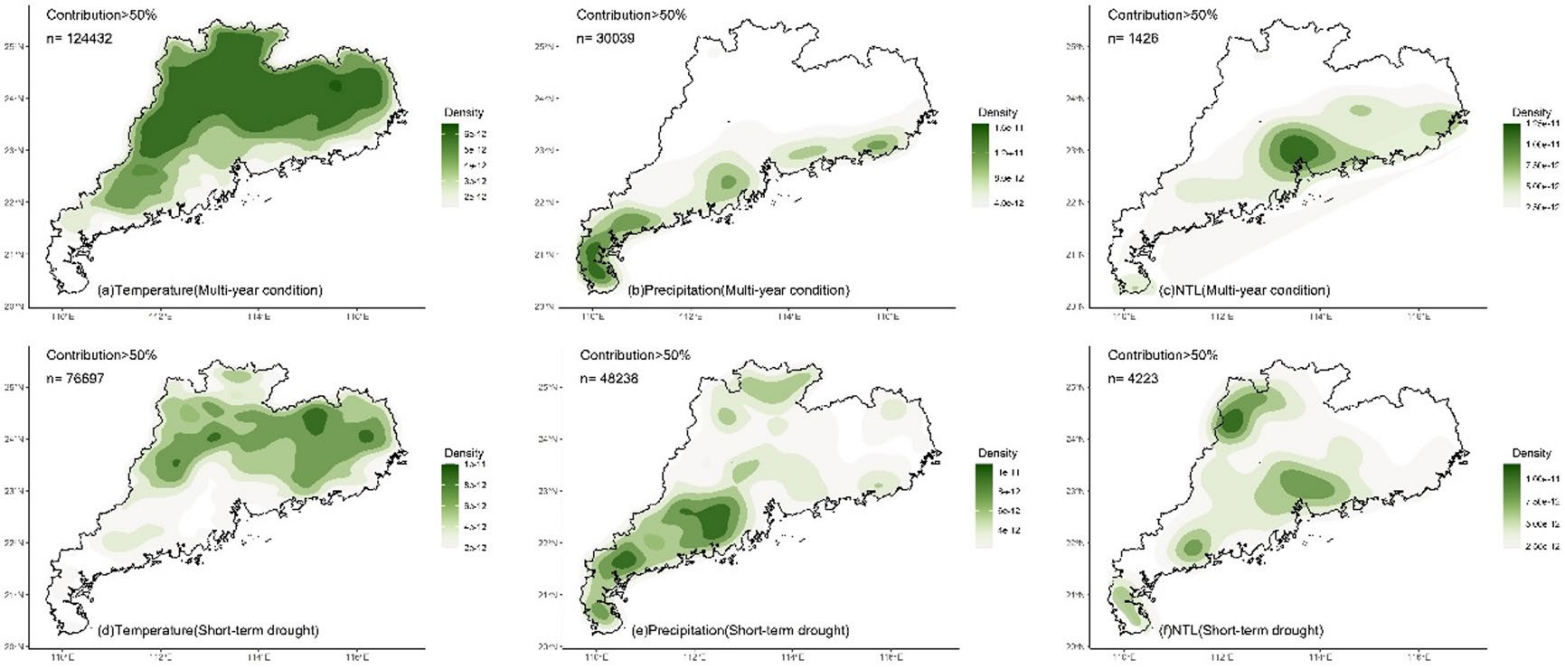
Kernel density of the contribution (> 50%, indicating that the variable is the dominant impact factor) of climate and human activities to NDVI under multi-year and short-term drought conditions. N represents the number of grids with the contribution greater than 50%.

Whether under multi-year or short-term drought conditions, the LMG contributions of climate and human activities to NDVI in different regions exhibited significant spatial differences (Fig. 3). For temperature, high contribution (> 50%) was mainly located in the northern and eastern regions (Fig. 3a and d) where temperature was the dominant impact factor. The southern region and Leizhou Peninsula had low contribution for temperature (< 50%). Compared to multi-year condition, under drought, the region with the high contribution of temperature decreased (Fig. 4a and d), and only the northern and eastern regions located in the north of the Tropic of Cancer maintained the high contribution. High contribution for precipitation (> 50%) was mainly located in the southern region and Leizhou Peninsula (Fig. 3b and e). Under drought, the contribution of precipitation increased, particularly in the western region where it became the dominant impact factor (Fig. 4b and e). NTL generally presented low contribution (Fig. 3c and f), while the contribution was higher (> 33%) in the urbanized region, especially in Pearl River Delta urban agglomeration (Fig. 4c and f). Compared to multi-year conditions, under drought, the impact of NTL increased with high contribution observed in many non-urbanized regions, such as the northern region and Leizho Peninsula (Fig. 4f). The contribution in urbanized regions, such as Pearl River Delta, also increased.

**Fig. 5:**
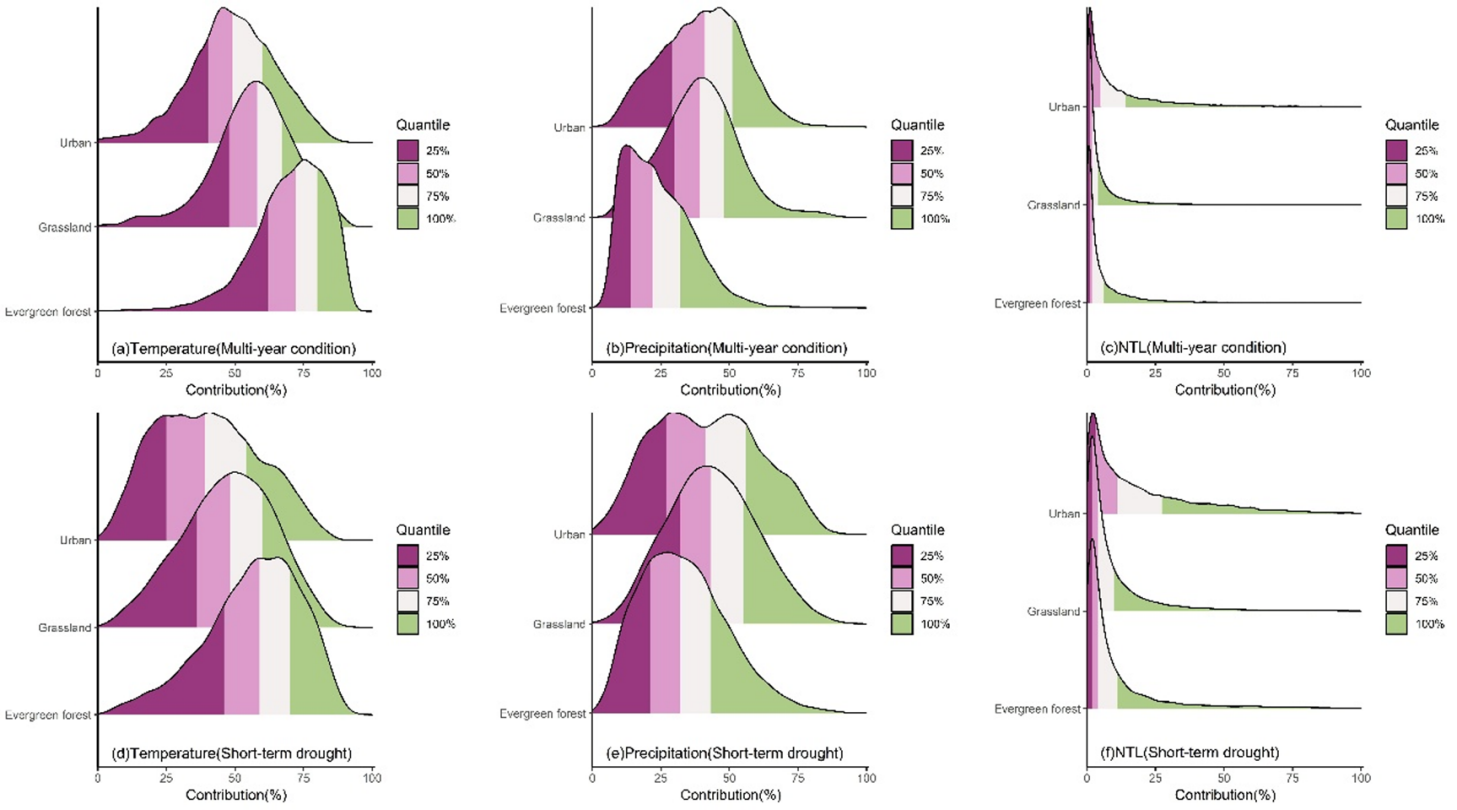
The ridgeline plot of the contribution of climate and human activities to the NDVI of land cover types without land cover change under different scenarios.

The LMG contributions of impact factors to NDVI changes exhibited significant differences across different land cover types (Fig. 5). For temperature, under multi-year conditions, temperature had the greatest impact on the NDVI of evergreen forests with the peak contribution mainly in the 72–80% range (Fig. 5a). The contribution distribution was more concentrated compared to grassland and urban region. For grassland, the peak of temperature contribution was mainly in the 48–67% range with the contribution range being more concentrated than that in urban region. In urban region, the contribution distribution was the most dispersed with the peak of temperature contribution mainly in the 40–60% range, significantly lower than that of natural vegetation. Under short-term drought, the distribution of temperature contribution for all land cover types shifted to the left (Fig. 5d) with the peak contribution decreasing to 59–70% for evergreen forest, 36–60% for grassland and 25–54% for urban region. Additionally, the contribution distribution became more dispersed with a significant change of distribution for urban region. For precipitation, under multi-year condition, the contribution of precipitation to the NDVI of evergreen forest was relatively low with the peak mainly in the 14–22% range (Fig. 5b), and the distribution was the most concentrated. The contribution of precipitation to the NDVI of grassland was mainly concentrated in the 30–48% range with a relatively concentrated distribution. In urban region, the precipitation contribution was similar to that of grassland ranging from 29 to 51%, and the distribution was the most dispersed. During short-term drought, the precipitation contribution for all land cover types shifted to the right (Fig. 5e), with peak values increasing to 21–32% for evergreen forest, 32–55% for grassland, and 27–56% for urban region. The distribution of contribution for evergreen forest and grassland became more dispersed, while the distribution in urban region shifted from a unimodal distribution to a bimodal distribution. For NTL, the distributions of contributions for all land cover types were highly concentrated at low values (Fig. 5c). The NTL contribution for natural vegetation such as evergreen forest and grassland was relatively small. The peak for evergreen forest was mainly in the 1–6% range, while grassland was concentrated in the 1–4% range. The NTL contribution to the NDVI of urban region was higher with the peak primarily in the 2–14% range. When short-term drought occurred, the contribution for all land cover types became more dispersed and increased (Fig. 5f). The peak for evergreen forest was mainly in the 2–11% range, and grassland was concentrated in the 2–10% range. The contribution for urban region increased the most reaching 3-27.2%.

**Fig. 6:**
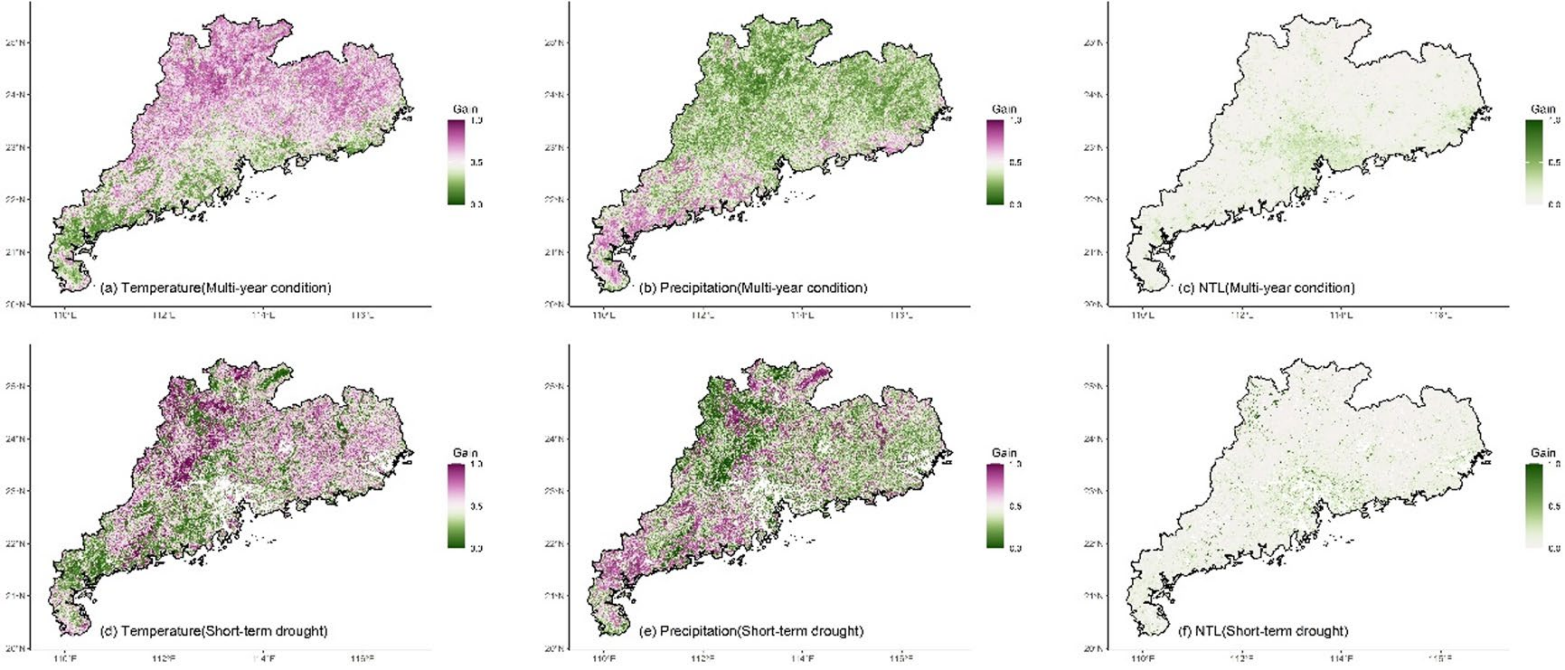
The XGBoost Gain of climate and human activities on NDVI under multi-year and short-term drought conditions. Gain represents the contribution of a variable to the prediction performance of model. The higher the Gain, the higher the contribution of the variable.

**Fig. 7:**
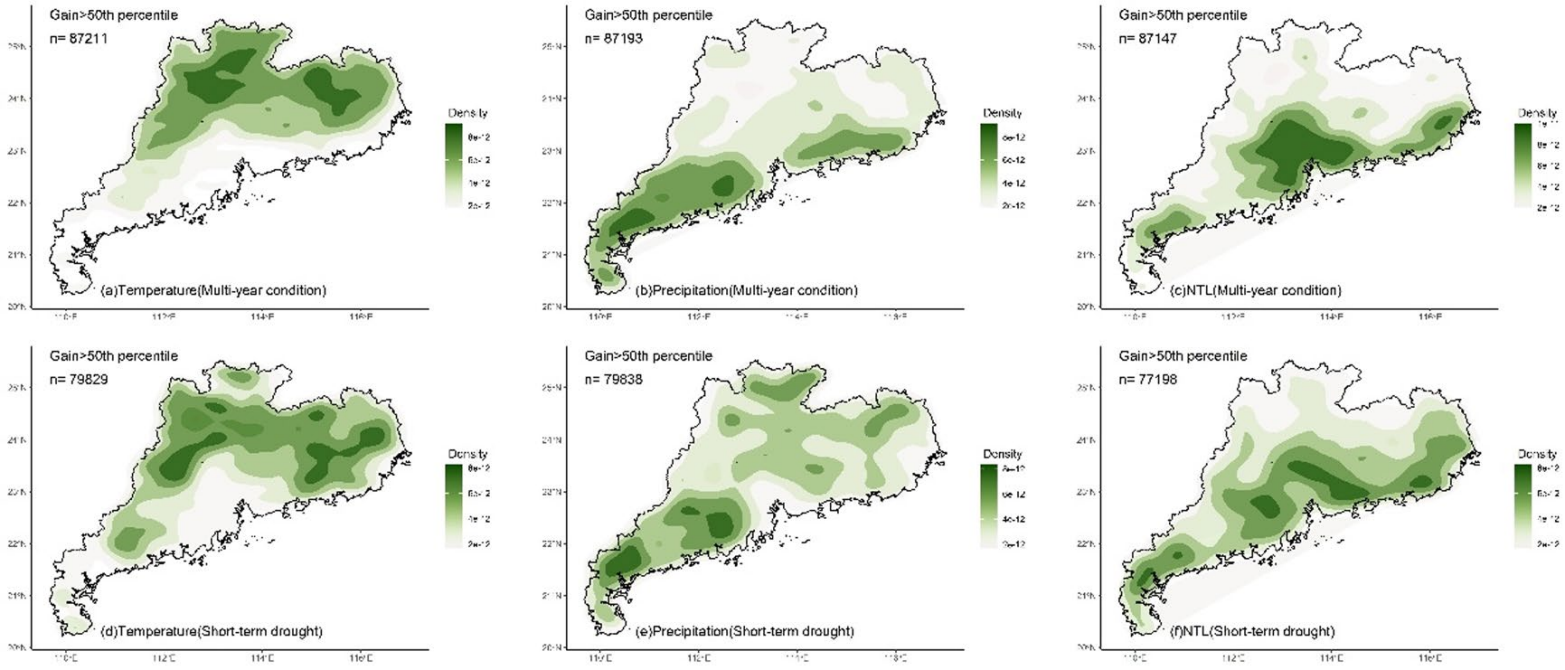
Kernel density of the XGBoost Gain (> 50th percentile, indicating that the variable is the dominant impact factor) of climate and human activities on NDVI under multi-year and short-term drought conditions. n represents the number of grids with the Gain greater than 50th percentile.

The temperature, precipitation and NTL contributions (Gain) to NDVI calculated by XGBoost exhibited spatial patterns similar to those of the LMG contributions (Fig. 6). For temperature, under multi-year condition, high Gain (> 0.5) of temperature-NDVI was mainly distributed in the northern and eastern regions located in the north of the Tropic of Cancer (Figs. 6a and 7a), while low Gain (< 0.5) was primarily concentrated in Leizhou Peninsula, the western and southern coastal regions. Under short-term drought, the regions with high Gain of temperature decreased (Figs. 6d and 7d). The northern and eastern regions still maintained high Gain, while Leizhou Peninsula, the western and southern coastal regions remained as low Gain regions. For precipitation, under multi-year condition, low Gain for precipitation-NDVI was mainly located in the northern and eastern regions (Fig. 6b), while high Gain was primarily distributed in Leizhou Peninsula, the western and southern coastal regions. During short-term drought, more high Gain regions appeared (Fig. 6d), particularly in the northern and eastern regions, where high Gain patches were observed (Fig. 7e). For NTL, under multi-year condition, high Gain for NTL-NDVI was mainly distributed in urbanized regions with distinct patches formed in the Pearl River Delta urban agglomeration (Figs. 6c and 7c). Urban regions in the eastern, western and northern Guangdong were also high Gain regions for NTL-NDVI. During short-term drought, high Gain located in the southern region (Figs. 6f and 7f).

**Fig. 8:**
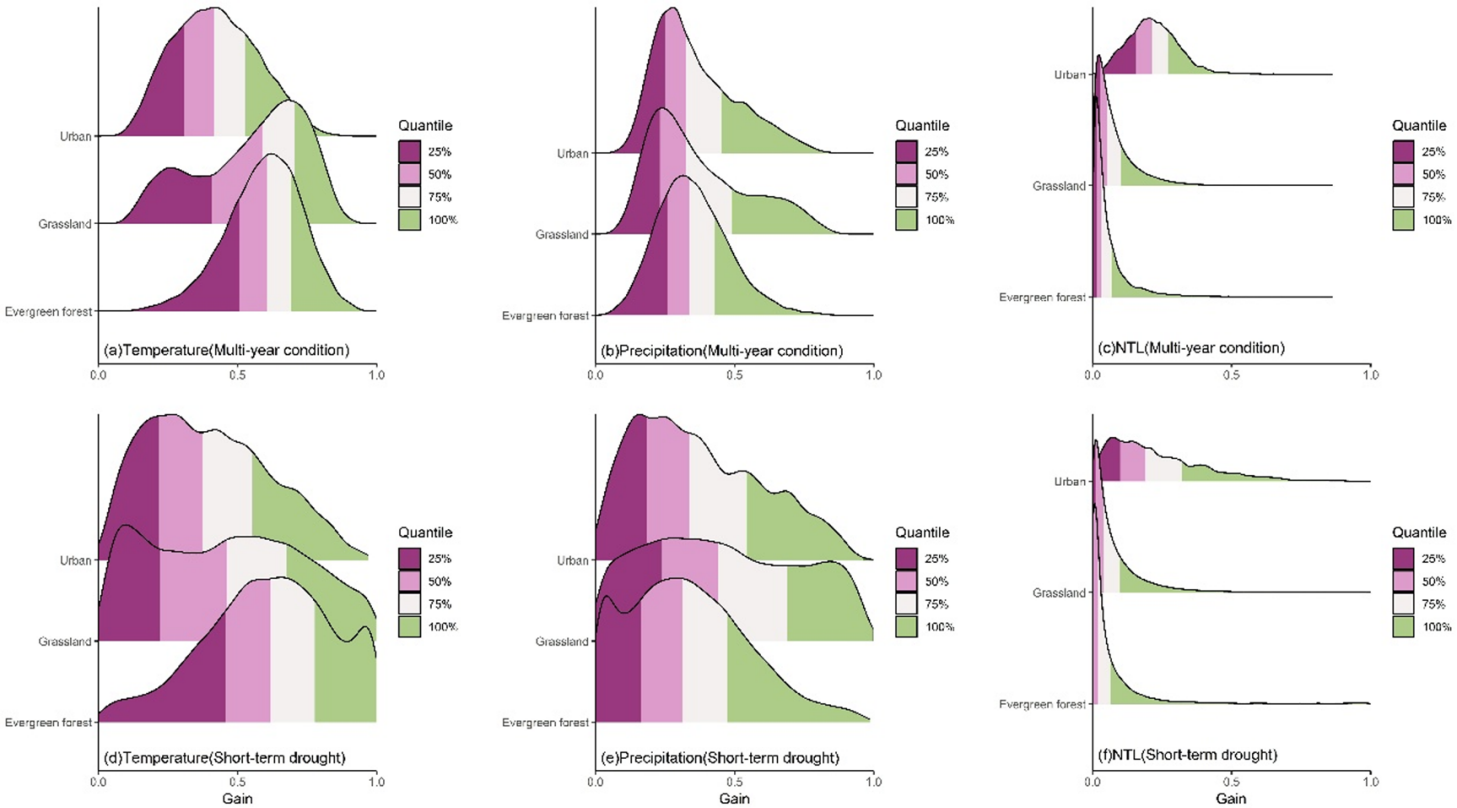
The ridgeline plot of the XGBoost Gain of climate and human activities on the NDVI of land cover types without land cover change under different scenarios. The higher the Gain, the higher the contribution of the variable.

The contributions of impact factors based on XGBoost Gain to NDVI change exhibited significant differences across different land cover types (Fig. 8). For temperature, under multi-year condition, the contribution of temperature to evergreen forest NDVI was the most concentrated with the Gain peak mainly in the 0.51–0.69 range (Fig. 8a). The distribution of Gain for grassland was more dispersed with the peak mainly in the 0.59–0.70 range, while the distribution for urban region was also more dispersed with the peak mainly in the 0.31–0.53 range. Under short-term drought, the Gain distribution became more dispersed (Fig. 8d), and the general distribution shifted to the left. The peak was mainly in the 0.46–0.78 range for evergreen forest and in the 0.22–0.68 range for grassland. The peak for urban region was mainly in the 0.22–0.55 range. For precipitation, under multi-year condition, the contribution of precipitation to NDVI for different land cover types exhibited a unimodal distribution (Fig. 8b). The Gain distribution for evergreen forest was the most concentrated, followed by urban region and grassland. mainly in the 0.26–0.43 range for evergreen forest and in the 0.23–0.49 range for grassland. For urban region, the Gain peak was in the 0.25–0.45 range. Under short-term drought, the Gain distribution also became more dispersed (Fig. 8e). The Gain peak for evergreen forest was mainly in the 0.16–0.47 range, while the Gain distribution for grassland was the most dispersed with the peak ranging from 0.23 to 0.44. For urban region, the peak was mainly in the 0.18–0.34 range. For NTL, the Gain distributions for all land cover types were highly concentrated at low values (Fig. 8c). The Gain for natural vegetation such as evergreen forest and grassland was relatively small with the peak for evergreen forest mainly in the 0.01–0.07 range and for grassland mainly in the 0.03–0.10 range. The NTL contribution to the NDVI of urban region was higher with the peak primarily in the 0.16–0.27 range. During drought, the Gain distributions for all land cover types became more dispersed (Fig. 8f). The peak was mainly in the 0.004–0.07 range for evergreen forest and in the 0.01–0.10 range for grassland. For urban region, the contribution increased the most, ranging from 0.10 to 0.32.

### The relationship among climate, human activities and vegetation greenness under different scenarios based on the linear mixed model

To further explore the positive or negative relationships between impact factors and vegetation greenness under drought, LMM was employed to establish a linear relationship between the impact factors and NDVI. Further, considering that droughts occur in different months and years and that NDVI levels vary across months and years (e.g., NDVI is generally higher in summer than in winter), month and year were treated as random effects in the LMM respectively. Additionally, the average effects of temperature, precipitation and NTL on NDVI were represented as fixed effects.

**Fig. 9:**
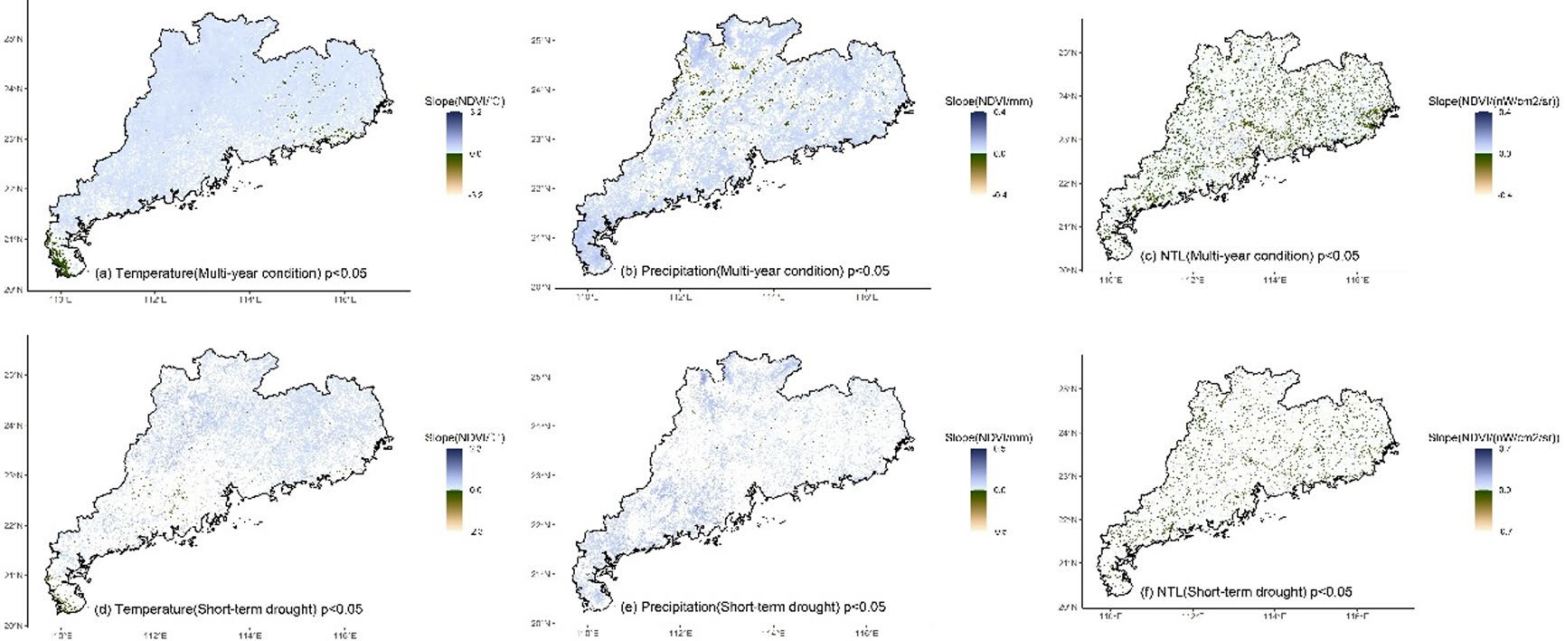
The slope for the impact of climate and human activities on NDVI based on LMM with month as a random effect under multi-year and short-term drought conditions.

**Fig. 10:**
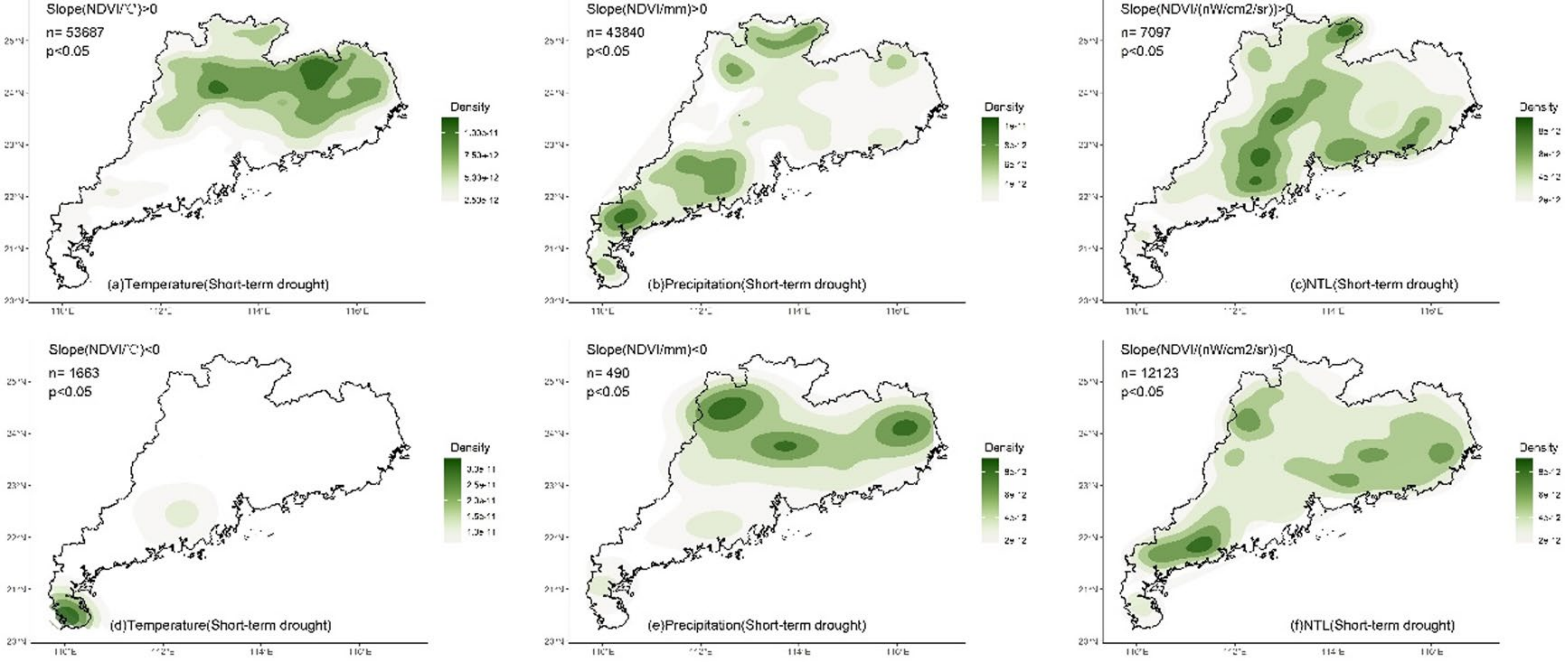
The slope for the impact of climate and human activities on NDVI based on LMM with month as a random effect under short-term drought conditions.

According to the LMM results that considered month as a random effect, most regions of Guangdong exhibited a significant positive slope between temperature and NDVI under multi-year condition (Fig. 9a), while Leizhou Peninsula and parts of the eastern region represented a significant negative slope. When short-term drought occurred, the regions influenced by temperature decreased, and the positive slope of temperature and NDVI disappeared in many regions, particularly in the west where it shifted from a positive to a negative slope (Fig. 9d). However, in the northern and eastern regions, temperature still exhibited a stable positive slope with NDVI (Fig. 10a), while the negative slope of temperature and NDVI remained stable in Leizhou Peninsula (Fig. 10d). Under multi-year condition, precipitation exhibited a significant positive slope with NDVI in most regions (Fig. 9b), while significant negative slopes were observed in parts of the northern and central regions. Under short-term drought, the impact of precipitation was predominantly characterized by positive slope (Figs. 9e and 10b), especially in the western region, and only a few northern and eastern regions exhibited negative slopes (Fig. 10e). Under multi-year condition, NTL showed a significant negative slope with NDVI in most regions (Fig. 9c). However, in urbanized regions, such as the Pearl River Delta and urban regions in the north, NTL exhibited a significant positive slope with NDVI. When short-term drought occurred, the impact of NTL increased. The Pearl River Delta and northern urban regions exhibited a positive slope clustering (Fig. 10f), while NTL had a significant negative impact on NDVI in the eastern and western regions (Fig. 10f).

**Fig. 11:**
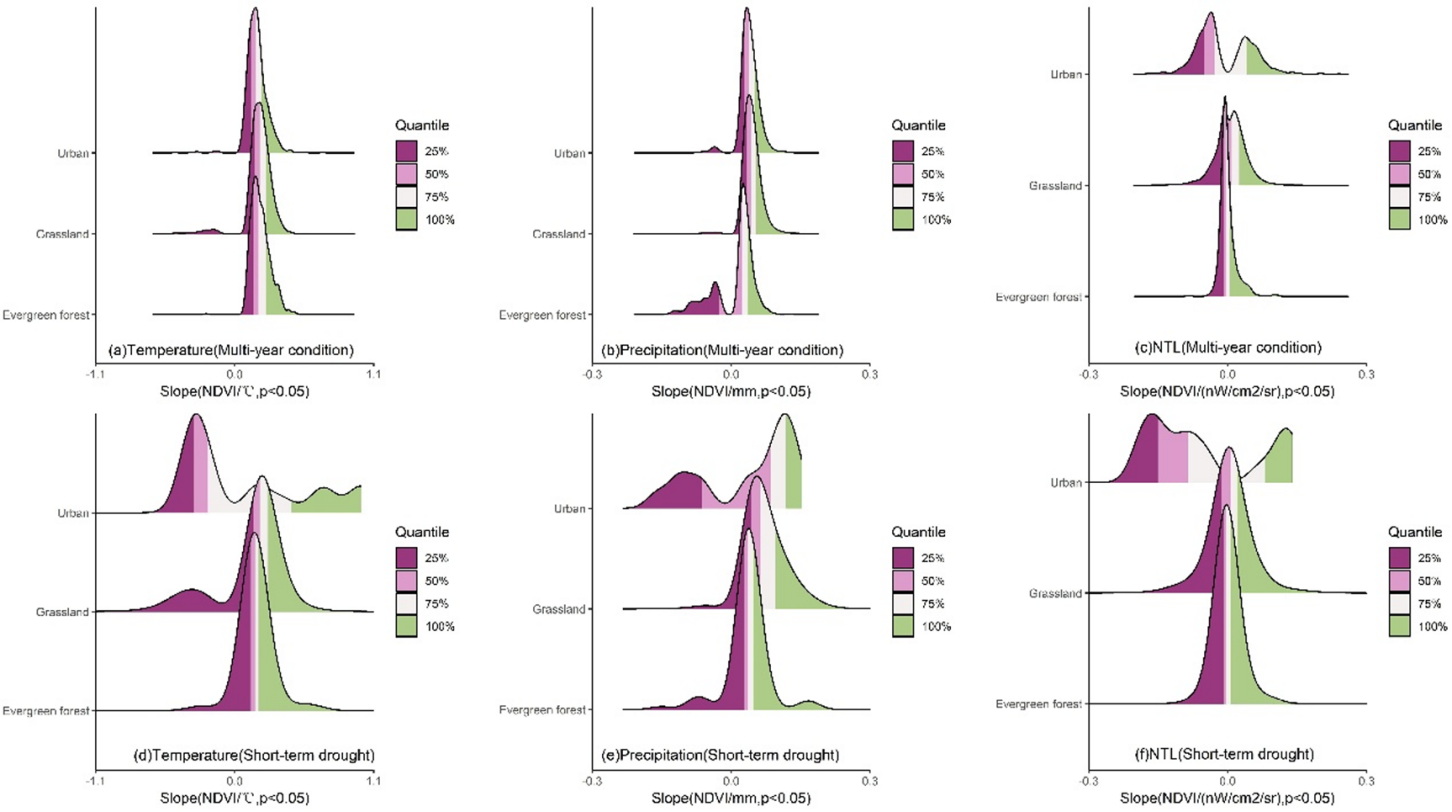
The ridgeline plot of the slope for the impact of climate and human activities on the NDVI of land cover types without land cover change based on LMM with month as a random effect under different scenarios.

The impact of multi-year temperature on NDVI predominantly exhibited a positive slope across all land cover types with the greatest impact on grassland (Fig. 11a), where the peak was mainly in the 0.16–0.25 range and the slope distribution was the most dispersed. This was followed by evergreen forest (0.15–0.25) where the slope distribution was the most concentrated, and urban region (0.13–0.21) which also exhibited a more dispersed slope. Short-term drought significantly changed the temperature-NDVI relationship for all land cover types with an overall shift to the left (Fig. 11d). The slope became more dispersed, especially in urban region and grassland where the distribution changed significantly. The peak was mainly in the 0.12–0.19 range for evergreen forest, in the 0.14–0.26 range for grassland and in the − 0.33 to 0.21 range for urban region. Slope values below the 50th percentile shifted from positive to negative, indicating that half of the urban region experienced a negative impact from temperature during short-term drought. For precipitation, the slope peak was mainly in the 0.02–0.04 range for evergreen forest (Fig. 11b), and the slope peak was concentrated in the 0.03–0.05 range for grassland, while for urban region the peak was in the 0.03–0.05 range. The slope distributions for grassland and urban region were relatively concentrated. Under short-term drought, the slope distributions for all land cover types shifted to the right. The peak was mainly in the 0.03–0.05 range for evergreen forest (Fig. 11e), in the 0.04–0.10 range for grassland and in the 0.08–0.12 range for urban region. The previously bimodal distribution of evergreen forest shifted to a unimodal distribution and the distribution for urban region changed from unimodal to bimodal. For NTL, under multi-year condition, the impact of NTL on evergreen forest NDVI was highly concentrated with the peak mainly in the − 0.01 to 0.005 range (Fig. 11c). The slope peak for grassland was mainly concentrated in the − 0.01 to -0.02 range, while the slope distribution for urban region exhibited a significant bimodal pattern with most of the slopes concentrated in the − 0.05 to -0.03 range. During short-term drought, the distribution became more dispersed (Fig. 11f). The peak was mainly in the − 0.01 to 0.01 range for evergreen forest, in the − 0.01 to -0.02 range for grassland, and in the − 0.1 to -0.09 range for urban region. Evergreen forest and grassland both exhibited unimodal distributions, while urban region exhibited a bimodal distribution with significant differences.

**Fig. 12:**
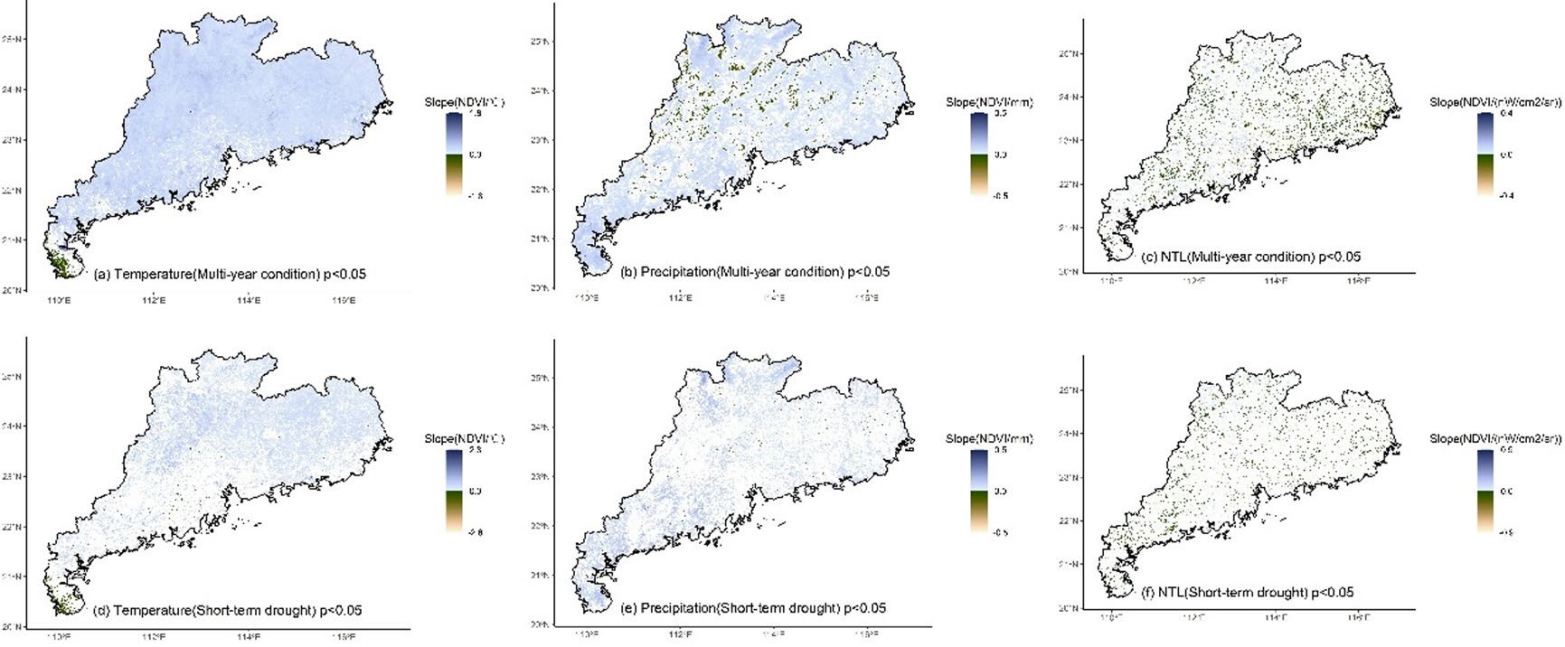
The slope for the impact of climate and human activities on NDVI based on LMM with year as a random effect under multi-year and short-term drought conditions.

**Fig. 13:**
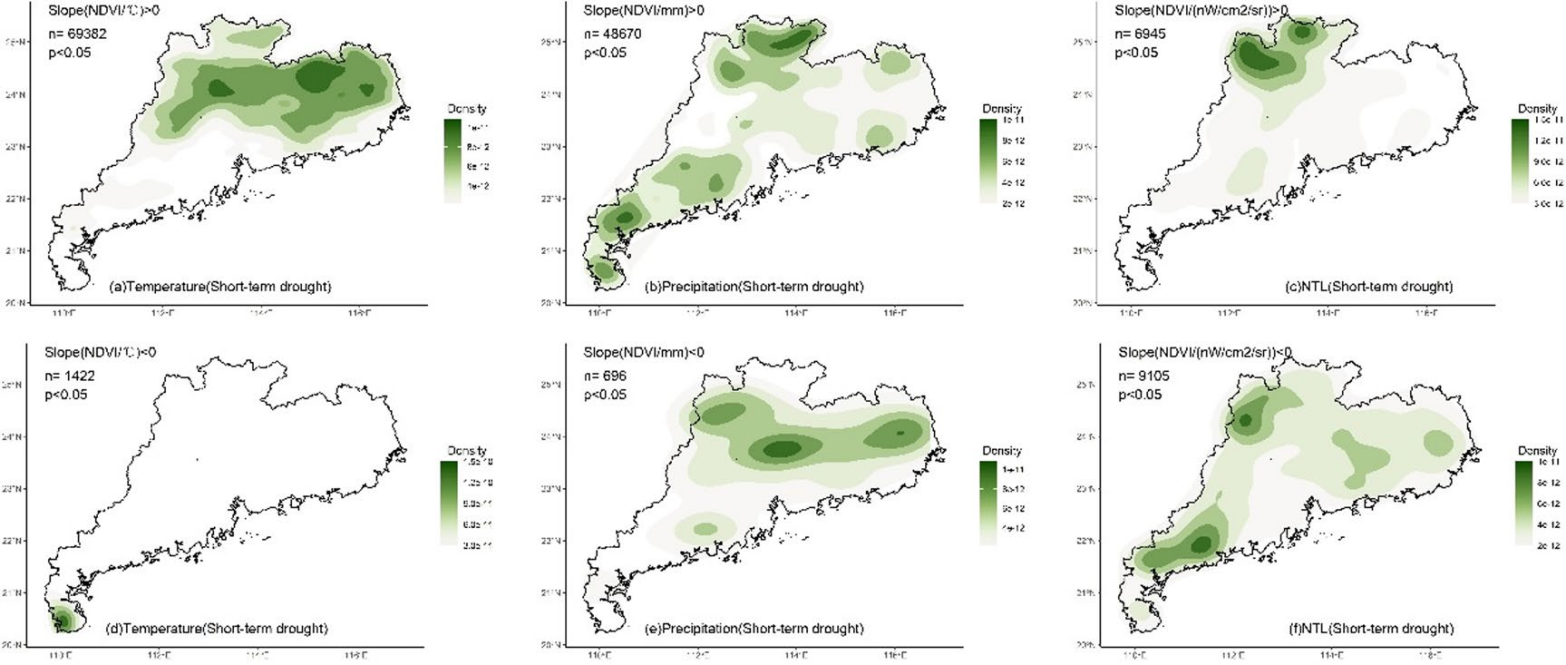
The slope for the impact of climate and human activities on NDVI based on LMM with year as a random effect under short-term drought conditions.

According to the LMM results that considered year as a random effect, multi-year temperature represented a significant positive slope with NDVI in most regions of Guangdong (Fig. 12a), while Leizhou Peninsula exhibited a significant negative slope. When short-term drought occurred, the regions influenced by temperature decreased and the positive slope relationship between temperature and NDVI disappeared in many regions, particularly in the west (Fig. 12d). However, in the northern and eastern regions, temperature still exhibited a stable positive slope with NDVI (Fig. 13a), while the negative slope between temperature and NDVI remained stable in Leizhou Peninsula (Fig. 13d). Under multi-year condition, precipitation showed a significant positive slope with NDVI in most regions (Fig. 12b), while significant negative slopes were observed in the north and part of the central region. Under short-term drought, the impact of precipitation was predominantly characterized by positive slopes (Fig. 12e), especially in the western region (Fig. 13b), while the previously negative slope in the northern and central regions shifted to positive and a small portion of northern and eastern regions exhibited a negative slope (Fig. 13e). Under multi-year condition, NTL represented a significant negative slope with NDVI mainly in the eastern region (Fig. 12c), while a significant positive slope was observed in the west and part of the northern region. During short-term drought, the northern region mainly exhibited positive slope (Fig. 13c). Additionally, the northern, western and eastern regions exhibited a negative slope (Fig. 13f).

**Fig. 14:**
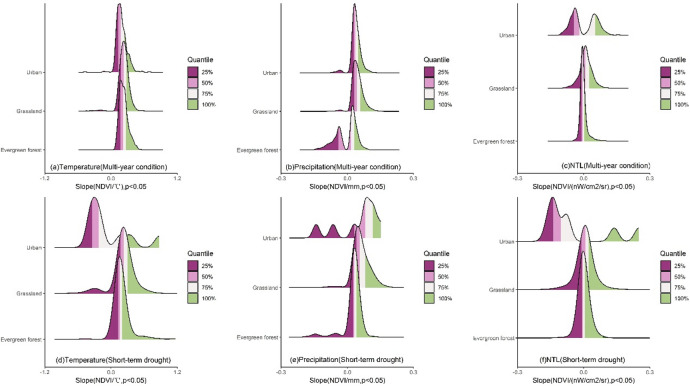
The ridgeline plot of the slope for the impact of climate and human activities on the NDVI of land cover types without land cover change based on LMM with year as a random effect under different scenarios.

The impact of multi-year temperature on NDVI predominantly represented a positive slope across all land cover types with the slope distribution being relatively concentrated (Fig. 14a). The greatest impact was observed on grassland with the peak mainly in the 0.19–0.28 range, followed by evergreen forest (0.17–0.27) and urban region (0.13–0.24). Short-term drought significantly altered the temperature-NDVI relationship for all land cover types with the slope becoming more dispersed (Fig. 14d). The distribution for evergreen forest and urban region shifted to the left, and the slope distribution for urban region changed significantly. The peak was mainly concentrated in the 0.13–0.20 range for evergreen forest, in the 0.17–0.30 range for grassland and in the − 0.34 to 0.17 range for urban region. The slope values below the 50th percentile shifted from positive to negative, indicating that half of the urban regions experienced a negative impact from temperature during drought. For precipitation, the slope peak for evergreen forest was mainly in the 0.02–0.03 range (Fig. 14b). The peak was concentrated in the 0.03–0.06 range for grassland and in the 0.03–0.05 range for urban region. The slope distributions for grassland and urban region were relatively concentrated, while that for evergreen forest was more dispersed. Under short-term drought, the slope distributions for all land cover types shifted to the right. The slope peak was mainly in the 0.03–0.04 range for evergreen forest (Fig. 14e), in the 0.04–0.08 range for grassland and in the 0.03–0.12 range for urban region. The previously bimodal distribution of evergreen forest became unimodal, while the slope distribution for urban region changed from unimodal to multimodal. For NTL, under multi-year condition, the impact of NTL on evergreen forest NDVI was highly concentrated with the peak mainly in the − 0.01 − 0.002 range (Fig. 14c). The slope peak for grassland was mainly concentrated in the − 0.01- -0.03 range, while the slope distribution for urban region exhibited a significant bimodal pattern with most of the slopes concentrated in the − 0.05- -0.02 range. During drought, the distribution became more dispersed (Fig. 14f). The peak was mainly in the − 0.01–0.01 range for evergreen forest, in the − 0.01- -0.02 range for grassland and in the − 0.1- -0.08 range for urban region. Both evergreen forest and grassland exhibited unimodal distributions, while urban region exhibited a multimodal distribution.

## Discussion

### The contributions of climate and human activities to vegetation is stable in the north of the tropic of cancer

The impact of multi-year climate on changes in vegetation greenness in Guangdong exhibited a stable spatial pattern. Both the linear LMG and nonlinear XGBoost methods detected essentially the same spatial distribution of the impacts of temperature and precipitation. In the northern (mainly north of the Tropic of Cancer) and eastern Guangdong, temperature was the dominant factor influencing vegetation greenness change (with a contribution greater than 50%, Figs. 3, 4 and 5). In the southern and western regions, precipitation was the dominant factor, especially in the southernmost Leizhou Peninsula. This stable spatial pattern indicates the fundamental and stable impact of climate on vegetation and reflects the differing demands for heat and moisture by vegetation in the northern and southern regions of the Tropic of Cancer^55^. For the relatively heat-abundant southern region, precipitation may be the main impact factor for vegetation growth, while temperature may play a dominant role in vegetation for the relatively heat-deficient northern region^56–58^. In addition, compared to the widespread impact of climate on vegetation greenness, the impact of human activities is highly concentrated, primarily in regions such as the Pearl River Delta urban agglomeration (Figs. 3, 4 and 5). In some urbanized regions, the impact of human activities on vegetation greenness even exceeds that of climate, indicating that urban vegetation requires special and separate analysis due to human intervention^59,60^.

Short-term drought altered the spatial pattern of impact of the factors in the south of the Tropic of Cancer. Under short-term drought, both LMG and XGBoost detected a significant weakening of impact of temperature on vegetation greenness, especially in the eastern and western regions. In the south of the Tropic of Cancer, the importance of precipitation significantly increased, indicating that drought made precipitation more important for vegetation in the eastern and western regions and water supply became more critical. This suggests that vegetation in the eastern and western regions is more vulnerable to climate change, and even short-term drought can alter the sensitivity of vegetation to climate, indicating that the resistance of these transitional zones to external environmental change is relatively low.

The impact of temperature and precipitation on vegetation in the northern region and Leizhou Peninsula remained stable (Figs. 3e and 6e). Short-term drought did not significantly alter the spatial impact of climate on vegetation greenness in these regions. The dominant impact factor in the northern region was still temperature, while precipitation remained the dominant factor in Leizhou Peninsula. For vegetation in the northern region, although drought occurred, the short duration of the drought made temperature the main limiting factor for vegetation growth. In the tropical Leizhou Peninsula, vegetation is highly dependent on precipitation due to the abundance of heat year-round. During drought, the importance of precipitation becomes even more pronounced. This high demand of water in tropical vegetation during drought is also observed in other tropical regions, such as India^61^, Southeast Asia^38^and Brazil^62^, indicating the high sensitivity of tropical vegetation to precipitation under drought.

The different responses of northern and southern vegetation to climate during short-term drought are partly attributable to latitudinal variations in thermal conditions. Compared with the tropical southern regions, regions in the north of the Tropic of Cancer experience relatively insufficient heat availability. In combination with the widespread distribution of mountainous terrain, this intensifies the thermal demand of northern vegetation such that a certain degree of warming may satisfy vegetation heat requirement^63,64^. Further, the large areas of evergreen forests in the northern region exhibit a certain resistance to drought. Their deeper root systems allow evergreen forests to access water from deeper soil layers to some extent which helps maintain growth during short-term drought^65^. Southern vegetation is predominantly grassland. Drought reduces the available water for shallow-rooted grasslands, thereby leading to a high contribution of precipitation^66^.

Additionally, short-term drought also altered the impact of human activities on vegetation greenness. Both LMG and XGBoost detected an increase in the impact of NTL on vegetation greenness (Figs. 3f and 6f), especially in non-urbanized regions where high contribution of NTL was observed. This indicates human intervention in vegetation during drought. However, compared to the dominant contribution of climate, the overall level of human intervention remains relatively small. Climate remains the dominant factor in variations in vegetation greenness during drought, which differs from regions in the northern drought areas where human activity is the dominant factor^67,68^. Moreover, compared to the significant spatial pattern of climate, the impact of human activities was primarily concentrated in urban region. This may be mainly due to human activities being influenced by various subjective factors, such as vegetation type, distance and ecological policy^23,69,70^. It is worth noting that the cumulative drought months are fewer in some regions (Fig. 2b), such as the northern region, which may lead to unstable variable contribution estimates in XGBoost due to the small sample size. Although both linear and nonlinear methods were used for cross-validation and yielded consistent conclusions, the risk of overfitting may still exist in certain regions.

### The relationship between temperature and vegetation greenness in the north of the tropic of cancer is stable under drought

After removing the monthly and inter-annual differences and fluctuations in vegetation greenness based on the LMM, the impacts of temperature, precipitation and NTL on NDVI were stable. Under multi-year condition, temperature exhibited a significant positive correlation with NDVI in most regions of Guangdong (Figs. 9 and 12). Considering the distribution of temperature contribution (Figs. 3 and 4), an increase in temperature leaded to a more significant increase in vegetation greenness in the northern and eastern regions. Leizhou Peninsula exhibited a significant negative correlation between temperature and NDVI, while the contribution of temperature in this region was relatively small. Therefore, the reduction in vegetation greenness caused by an increase in temperature was limited. For the precipitation-NDVI relationship, with the exception of some areas in the northern region where there was a negative correlation, the remaining regions exhibited a significant positive correlation (Figs. 7 and 9). Considering the distribution of precipitation contribution (Figs. 3 and 4), an increase in precipitation leaded to a greater increase in vegetation greenness in the western region and Leizhou Peninsula, while the increase was smaller in the northern and eastern regions. The relationship between human activities and vegetation greenness represented a positive correlation in the Pearl River Delta urban agglomeration indicating that vegetation in this region was relatively more positively influenced by human (Fig. 10). For the vegetation in most of the eastern and western regions, human activities had an adverse impact.

Short-term drought generally altered the spatial patterns of the impact factors on vegetation greenness to some extent. Drought significantly reduced the regions where temperature and NDVI were positively correlated (Figs. 10 and 13), particularly in the western region. In some regions, the correlation shifted to negative with temperature increasing from promoting vegetation greenness to inhibiting its increase. However, the northern and eastern regions maintained a positive correlation, indicating the stability of temperature-NDVI relationship in these regions. Further, temperature continued to maintain a high contribution during drought in the northern region, indicating that an increase in temperature would lead to a greater increase in vegetation greenness in this region. The relationship between precipitation and vegetation greenness was generally positive with previously negative correlation shifting to positive under short-term drought. Additionally, due to the increased contribution of precipitation in the eastern and western regions (Fig. 3), the increase in precipitation in these regions will lead to a greater increase in vegetation greenness.

Short-term drought altered the relationship between regional climate and vegetation to some extent. This reconstruction of the relationship often indicates the vulnerability of the vegetation and its ecosystem in that region^71,72^, suggesting that vegetation is unable to maintain its original relationship with climate through its own mechanisms under the pressure of climate change. In contrast, regions where the climate-vegetation relationship remains stable have a certain degree of resilience in their vegetation and ecosystem which can adapt to climate change through self-regulation^73,74^. For example, vegetation continued to show a positive correlation with temperature under short-term drought condition in the northern part of Guangdong, indicating that the vegetation in this region has some degree of adaptation to high temperature. In contrast, the positive correlation between temperature and vegetation greenness in the western region disappeared under the disturbance of drought, and some regions even shifted to a negative correlation. This indicates that vegetation in this region is highly sensitive to temperature change and has difficulty maintaining its original climate response mechanism, reflecting the ecological vulnerability of the region.

### Short-term drought alters the sensitivity of vegetation greenness to climate and human activities across different land cover types

The results from both LMG and XGBoost indicated that short-term drought reshaped the dominant structure of contributions of climate and human activities to vegetation greenness across different land cover types (Figs. 5 and 8). For evergreen forest, the contribution of temperature was the highest under multi-year condition (Fig. 5a), while drought caused the peak contribution to decrease by approximately 10–13% and the contribution distribution shifted to the left (Fig. 5d). This indicates that under drought scenarios, the role of temperature in evergreen forest weakens and the spatial heterogeneity of temperature contribution increases with the contribution becoming more dispersed. In addition, drought increased the contribution of precipitation to evergreen forest vegetation greenness as indicated by the rightward shift of the peak (Fig. 5b and e), and the peak contribution increased by approximately 7–10%. This indicates that the impact of precipitation increases and the water limitation for evergreen forest strengthens. The contribution of human activities remained relatively low (Fig. 5c and f), while drought slightly increased the impact of human intervention with the peak contribution rising by approximately 1–5%. However, the overall impact remained small. Grassland exhibited a situation similar to that of evergreen forest with the contribution of temperature being the highest among the impact factors under multi-year condition (Fig. 5a). Drought weakened this contribution with the peak contribution decreasing by approximately 7–12% (Fig. 5d) and the contribution distribution shifted to the left. This indicates a reduction in the impact of temperature and an increase in the spatial heterogeneity of response of grassland to temperature. Meanwhile, the peak contribution of precipitation increased by approximately 2–7%, indicating that the sensitivity of grassland to moisture increases under short-term drought. Additionally, the contribution of human activities was relatively low overall. Although drought leaded to an increase in the peak contribution of human activities by approximately 1–6%, the overall impact was still quite small. For urban region, drought caused a significant decrease in the peak contribution of temperature by approximately 6–15%, while the contribution of precipitation exhibited a bimodal distribution indicating significant differences in the impact of precipitation across different cities. The impact of human activities was more pronounced in urban region, and drought significantly increased the impact of human activities with the peak contribution rising by approximately 1-13.2%.

The results from both LMM scenarios were similar indicating that the relationship among temperature, precipitation, NTL and NDVI across different land cover types was stable (Figs. 11 and 14). Under multi-year condition, temperature and precipitation were generally positively correlated with all land cover types. NTL remained negatively correlated with vegetation greenness in most urban region and the slope distribution exhibited a bimodal pattern, indicating significant variation in human intervention on vegetation across different cities. The relationship between NTL and the vegetation greenness of evergreen forest and grassland exhibited a complex pattern with peak slopes representing both negative and positive correlations (Figs. 11c and 14c). Short-term drought caused the slope distribution of vegetation greenness and temperature for all land cover types to shift to the left. Although temperature continued to show a positive correlation with natural vegetation, the slope values decreased indicating a reduction in the impact of temperature. Drought significantly altered the positive correlation between temperature and vegetation greenness in urban region with the positive correlation turning negative in half of the cities (Figs. 11a and d and 14a and d). Under short-term drought, the slope distribution of precipitation and vegetation greenness for all land cover types shifted to the right overall indicating an increase in the impact of precipitation (Figs. 11b and e and 14b and e). In urban region, the slope distribution became multimodal indicating significant heterogeneity between different cities. Further, drought caused the slope distribution of vegetation greenness and NTL for all land cover types to become more dispersed (Figs. 11c and f and 14c and f). This suggests that even within the same land cover type, the internal differences increase under drought conditions. In urban region, the peak slopes became negative and their absolute values increased indicating that drought deepens human intervention in urban vegetation and reduces vegetation greenness. It suggests that the allocation of urban water resources under water scarcity may be involved^75,76^. Changes in water usage behavior may have resulted in inadequate water allocation for urban vegetation which could be explored in the future by incorporating water supply management data. Meanwhile, because NTL is often associated with impervious surfaces and the urban heat island effect, the combination of these factors may increase drought stress, leading to greater vegetation sensitivity. Therefore, the impact of NTL may involve multiple underlying mechanisms. Additionally, the limitations of NTL data may also introduce certain errors. Although this paper used NTL as a proxy for human activities to analyze their spatial distribution, as in other studies^31,32^, and employed VNP46A3 which has undergone cloud screening, atmospheric and moonlight BRDF correction^77,78^, data errors were still inevitably present. Moreover, while NTL is generally positively correlated with human activities associated with nighttime lighting, it does not fully reflect all dimensions of human activity. For example, in forested areas, NTL values are typically low, but this does not necessarily mean the absence of human activities. Small areas with high NTL value may correspond to settlements or infrastructure in forested areas indicating potential human disturbances in these regions.

Consistent with the results of other studies on the impacts of drought on different land cover types^79–81^, the results of this paper also represented that different land cover types in subtropical humid region exhibited distinct response patterns to short-term drought. For example, the contributions of temperature and precipitation to evergreen forest changed the most during drought, indicating that evergreen forest are more sensitive to climate change during drought compared to grassland and urban region. However, because the evergreen forest in Guangdong is mainly located in the northern region, even though drought weakens the impact of temperature, temperature still plays a dominant role in evergreen forest, while it is not always dominant for grassland and urban region. This indicates that the original energy-water synergy mechanism is relatively stable for evergreen forest^82,83^. In addition, due to recent ecological policies^84^, evergreen forest and grassland are less disturbed by human activities and their vegetation greenness changes are mainly influenced by climate. This is different from the pattern observed in urban vegetation greenness change. Additionally, some urban grids may contain a mixture of vegetation and impervious surfaces, causing the NDVI of vegetation to be diluted by impervious surfaces and thereby biasing the representation of vegetation conditions. Moreover, because impervious surfaces exhibit little response to climate change, they may reduce the magnitude of NDVI responses to changes in climatic variables, leading to an underestimation of vegetation sensitivity to climate^85–87^.

## Conclusions

To explore the sensitivity of vegetation greenness to climate and human activities and its resilience to short-term drought in subtropical humid region, this paper used SPEI-3 to identify short-term drought events (less than 6 months) in Guangdong. LMG and XGBoost were employed to analyze the contribution and spatial pattern of temperature, precipitation and NTL to the NDVI of evergreen forest, grassland and urban region. Further, LMM was used to examine the linear relationships and corresponding spatial distributions among temperature, precipitation, NTL and the NDVI of different land cover types with month and year considered separately as random effects. All analyses were conducted under both multi-year and short-term drought scenarios. This paper helps to explain the impacts of climate change and human activities on subtropical vegetation during drought, providing a scientific basis for climate adaptation strategies and for assessing the environmental impacts of human activities. The main conclusions are drawn:

1. Under short-term drought, the regions with high temperature contribution decreased significantly, especially in the south of the Tropic of Cancer. However, temperature maintained a stable dominant influence and short-term drought was insufficient to alter the stable relationship between vegetation and temperature in the heat-limited northern region of the Tropic of Cancer. Meanwhile, the contribution of precipitation increased significantly in the eastern and western regions. Drought also leaded to an increased contribution of NTL in non-urbanized regions and made NTL the dominant factor for NDVI changes in some urban regions, such as parts of the Pearl River Delta.
2. Short-term drought leaded to a decrease in the contribution of temperature to the NDVI of all land cover types, while the contribution of precipitation increased. The temperature contribution of evergreen forest decreased the most (10–13%), followed by urban region (6–15%) and grassland (7–12%). The precipitation contribution increased by 7–10% in evergreen forest and by 2–7% in grassland, while urban region exhibited a bimodal distribution of precipitation contribution indicating significant differences in the impact of precipitation across different cities. The contribution of NTL also increased, while the change was relatively small for vegetation (1–5%). In urban region, the contribution of NTL significantly increased (1-13.2%). Additionally, drought caused the distribution of contribution across all land cover types to become more widespread, indicating an increased spatial heterogeneity in the sensitivity of vegetation to climate and human activities.
3. In most parts of Guangdong, temperature and precipitation exhibited significant positive correlations with NDVI over multiple years. The positive correlation between temperature and NDVI in the northern region, located in the north of the Tropic of Cancer, persisted under drought. However, drought weakened the positive temperature-NDVI correlation in the western region, located in the south of the Tropic of Cancer, while strengthening the positive impact of precipitation. Drought made the positive correlation between NTL and NDVI to be primarily concentrated in urbanized regions, while a negative correlation located in the eastern and western regions.
4. Short-term drought altered the relationship among climate, human activities and the vegetation greenness of different land cover types. Under drought condition, although temperature still maintained a positive correlation with the NDVI of vegetation, the strength of this correlation decreased. In more than half of the urban regions, the temperature-NDVI relationship shifted from positive to negative. Drought enhanced the positive impact of precipitation on the NDVI of all land cover types. The impact of NTL on vegetation remained relatively stable with the increased spatial heterogeneity. Drought also strengthened the negative impact of NTL on urban NDVI, potentially due to the conflict of water resource allocation under water scarcity.

## Data Availability

The datasets used and/or analyzed during the current study are available from the corresponding author on reasonable request.
